# Development of Viral Vectors for Gene Therapy for Chronic Pain

**DOI:** 10.1155/2011/968218

**Published:** 2011-04-07

**Authors:** Yu Huang, Xin Liu, Lanlan Dong, Zhongchun Liu, Xiaohua He, Wanhong Liu

**Affiliations:** ^1^School of Medicine, Wuhan University, Donghu Road #185, Wuchang, Wuhan 430071, China; ^2^College of Pharmacy, Wuhan University, Donghu Road #185, Wuchang, Wuhan 430071, China; ^3^Research Center of Food and Drug Evaluation, Wuhan University, Donghu Road #185, Wuchang, Wuhan 430071, China

## Abstract

Chronic pain is a major health concern that affects millions of people. There are no adequate long-term therapies for chronic pain sufferers, leading to significant cost for both society and the individual. The most commonly used therapy for chronic pain is the application of opioid analgesics and nonsteroidal anti-inflammatory drugs, but these drugs can lead to addiction and may cause side effects. Further studies of the mechanisms of chronic pain have opened the way for development of new treatment strategies, one of which is gene therapy. The key to gene therapy is selecting safe and highly efficient gene delivery systems that can deliver therapeutic genes to overexpress or suppress relevant targets in specific cell types. Here we review several promising viral vectors that could be applied in gene transfer for the treatment of chronic pain and further discuss the possible mechanisms of genes of interest that could be delivered with viral vectors for the treatment of chronic pain.

## 1. Introduction

Chronic pain is defined by the U.S. Food and Drug Administration as pain that persists for more than 3 months [1]. This leads to great suffering for patients and results in a heavy burden for society. Chronic pain is not simply related to anatomical reorganization; other changes in neurotransmission and electrophysiological activity are also involved in the pain pathway [2]. Conventional drug treatment has many limitations, such as drug dependence, tolerance, respiratory depression, and other systemic side effects. The development of gene therapy has opened the possibility of using either nonviral or viral vectors to transduce genes encoding antinociceptive substances to treat chronic pain. Compared with nonviral systems, viral vectors are much more efficient in delivering exogenous genes to target cells and inducing long-term gene expression [3]. However, not all viruses are suitable for gene delivery. For instance, murine leukemia virus and lentivirus are both retroviruses, but lentivirus can infect nondividing cells, while murine leukemia virus cannot. This is the reason murine leukemia virus is usually not used as a gene carrier in neurological disease treatments [4]. It was reported that 2 of 11 children receiving gene therapy using a retroviral vector caused leukemia. A possible reason is the integration of viral gene may activate oncogenes. So the safety of retroviral vectors has been paid more and more attention [5, 6]. The ideal gene therapy vector should not be able to replicate its own DNA and be conducive to long-term gene expression. In addition, it should be nonpathogenic and nontoxic [7]. Viral vectors are generally created by deleting nonessential genes from the virus while retaining the structural motifs necessary to transfer its genome into the host [8]. Herpes simplex virus, adeno-associated virus, adenovirus, lentivirus, and human foamy virus can all be used as viral vectors for gene therapy for chronic pain. These viral vectors are most commonly used because of their low immunogenicity, natural integration ability and other features. What's more, they can infect both dividing and nondividing cells. In this paper, published data about these vectors will be reviewed, and their applications in gene therapy of chronic pain will be discussed.

## 2. Herpes Simplex Virus (HSV)

Herpes simplex virus has neuronotropic features and naturally maintains lifelong residency in the nucleus of infected neurons, making it suitable for transduction in the nervous system [9]. Primary afferent neurons are the natural targets of HSV. HSV vector infects epithelial cells or neurons directly after subcutaneous injection or topical application, then penetrates peripheral afferent nerve terminals, and is retrogradely transported to cell bodies in dorsal root sensory ganglia [10]. For the successful use of HSV vectors, it is important to reduce their toxicity, modulate the time course of transgene expression, and precisely target specific cells [11]. A number of different HSV-based nonreplicative vectors with reduced cytotoxicity have been developed for specific gene therapy applications [11, 12]. Recombinant replication-defective HSV vectors expressing opioid peptides or glutamic acid decarboxylase could inhibit nociceptive neurotransmission at the first synapse between primary nociceptive and second-order neurons in the dorsal horn of the spinal cord [1, 13]. These viral vectors have been shown to have analgesic effects in models of inflammatory pain, neuropathic pain and cancer-related pain [1, 13, 14]. The different applications of HSV vectors for gene therapy of chronic pain are summarized in Table 1.

Endogenous opiate peptides acting on synapses in the dorsal horn of the spinal cord inhibit transmission of nociceptive stimuli [15]. Wilson et al. firstly demonstrated the antihyperalgesic potency of HSV-mediated preproenkephalin expression in DRG in a model of acute pain [16]. In a model of ongoing polyarthritis-associated inflammatory pain, Braz et al. enhanced enkephalin synthesis in sensory neurons of polyarthritic rats and reduced hyperalgesia by using recombinant HSV containing rat preproenkephalin A cDNA [17]. It has been reported that application of HSV vectors encoding preproenkephalin (PPE) or endomorphin-2 (EM-2) can lead to an antiallodynic effect in both rat and monkey models of chronic pain [9, 18–20]. Administration of the opioid receptor antagonists naloxone and naloxone methiodide can block this analgesic effect [21], suggesting that endogenous opioid peptides reduce pain perception through both central and peripheral opioid receptors. In addition, animals did not develop tolerance to the continued production of vector-mediated enkephalin over a period of several weeks [22]. 

Hao et al. demonstrated that expression of interleukin-4 (IL-4) in dorsal root ganglion (DRG) neurons achieved via HSV-mediated gene transfer in vivo reduced mechanical allodynia and thermal hyperalgesia in a spinal nerve ligation (SNL) model of neuropathic pain. However, it did not prevent the ultimate development of neuropathic pain [23]. Another cytokine, tumor necrosis factor-*α* (TNF-*α*), is overexpressed by activated microglia and is correlated with the emergence of mechanical allodynia [24]. Thus, HSV vector-mediated gene transfer of the p55 TNF soluble receptor (sTNFRs) could reduce pain, diminish the expression of mTNF-*α*, and decrease the number of ED1-positive cells as well as phosphorylation of p38 MAP kinase (p-p38) in the dorsal horn. This suggests that sTNFR may block the TNF-*α* signal after injury and reduce pain-related behavior [24]. 

Transfer of the gene that encodes glutamic acid decarboxylase (GAD67) into DRGs using a replication-defective HSV vector could reduce neuropathic pain [25, 26]. This may be because subcutaneous inoculation of HSV vector promotes the release of gamma aminobutyric acid (GABA), which inhibits nociceptive neurotransmission [25, 26]. Increased expression of the Na_v_1.7 sodium channel in sensory neurons occurs after peripheral inflammation and potentially increases neuronal excitability. Application of a recombinant HSV vector encoding an antisense sequence to the Na_v_1.7 gene could produce a long-lasting or even permanent decrease in inflammatory pain and hyperalgesia in treated tissue without affecting untreated tissue [27].

## 3. Adeno-Associated Virus (AAV)

Adeno-associated viral vectors are commonly used to deliver therapeutic genes to target tissues because of their low immunogenicity [28]. AAV2 is the most widely used serotype in gene therapy, while other new efficient AAV vector types such as AAV8 are under development. Recombinant AAV can insert up to 6 kb of foreign DNA into the host genome [29]. AAV enters the cell through the internalization of clathrin-coated pits and escapes endosomal degradation via acidification of the late endosome [30].

Spinal cord glia and glial proinflammatory cytokines contribute to the initiation and maintenance of neuropathic pain. This suggests that targeting glial activation or suppressing proinflammatory cytokines may be an effective therapeutic strategy. Intrathecal administration of an AAV2 vector encoding an anti-inflammatory cytokine (IL-10) can reverse neuropathic pain because IL-10 suppresses the production of proinflammatory cytokines and also antagonizes the signaling pathway activated by these cytokines [31]. Delivery of recombinant AAV encoding a small hairpin RNA against GTP cyclohydrolase I (rAAV-shGCH1) into DRG neurons can also relieve neuropathic pain through downregulation of GTP cyclohydrolase I (GCH1) levels. This may occur because GCH1 downregulation leads to decreased microglial activation in the dorsal horn, implying that transcriptional activation of GCH1 in the DRG is associated with the development of pain and inflammation [32].

Increased *μ*-opioid receptor expression in DRG neurons achieved by recombinant AAV-mediated gene transfer enhanced the antinociceptive effects of morphine in rats. The limitation of this study is that direct injection of recombinant AAV into the DRG can lead to tissue damage [33]. Storek et al. found that a self-complementary recombinant adeno-associated virus serotype 8 (sc-rAAV8) expressing the analgesic gene prepro-*β*-endorphin (pp*β*EP) led to significant reversal of mechanical allodynia, and this antiallodynic effect could be reversed by application of the *μ*-opioid antagonist naloxone [34]. 

The levels of neurotrophins in the spinal cord have been proposed to restore normal function after nervous system injury. Intraspinal rAAV-mediated overexpression of brain-derived neurotrophic factor (BDNF) reduces allodynia and hyperalgesia induced by chronic constriction injury (CCI) [35]. However, direct spinal application of BDNF contributes to mechanical hypersensitivity and neuropathic pain via activation of spinal microglia [36]. Microglial-derived BDNF then mediates central sensitization by attenuating inhibitory synaptic transmission [37]. The exact effect of BDNF in the mechanism of pain still requires further clarification.  Table 2 presents examples of AAV vector-mediated gene transfer in pain models. 

Efficient and long-term gene transfer in the white matter of the spinal cord, DRG neurons, and peripheral nerves can be mediated by intraperitoneal or intramuscular injection of AAV [38]. However, some important issues for gene therapy still need to be explored. First, the extent of transgene expression in the DRG following intrathecal injection is unknown. Second, no studies have demonstrated the effects of the vector on the brain if the vector is injected at thoracic levels of the spinal cord or higher. Third, the potential for infection of other organs beyond the nervous system after intrathecal injection of AAV requires further examination [39].

## 4. Adenovirus (AV)

Adenoviral vectors have a gene carrying capacity of 7.5 kb and can transduce both dividing and nondividing cells [4]. These viral vectors cannot integrate into the host genome, so there is a low risk of insertional mutagenicity [40]. The first adenoviral vectors were constructed by substituting the viral early gene 1 (E1) with a therapeutic gene. The E1 gene is essential for adenoviral replication, so modification of the adenoviral genome through the deletion of E1 creates a replication-defective vector and provides sufficient space for foreign gene insertion [8]. More efficient gene carriers were obtained by altering more genes in the viral genome, such as E2, or using polyethylene glycol (PEG) to facilitate transfer [29, 41]. Adenoviruses have greater transgenic capacity if more viral genes are removed [29, 41].

Loss of GABAergic inhibitory interneurons in the superficial dorsal horn of the spinal cord reduces GABAergic tone and contributes to neuropathic pain after spinal cord injury [42]. Vit et al. constructed a serotype 5 adenovector to transfer the glutamic acid decarboxylase (GAD65) gene into satellite glial cells (SGCs) of the trigeminal ganglion. They found that GAD65 expression in the trigeminal ganglion led to an analgesic effect via increased GABA synthesis. This analgesic effect could be blocked by selective GABA_A_ receptor antagonists but not by an antagonist of GABA_B_ receptors [43]. In an inflammation model of persistent pain, administration of a recombinant adenovirus encoding endogenous opioid *β*-endorphin into the cerebrospinal fluid (CSF) surrounding the spinal cord attenuated inflammatory hyperalgesia but had no effect on basal nociceptive responses [44].

Some anti-inflammatory cytokine genes can also be transduced by adenoviral vectors to attenuate chronic pain. Spinal cord glial cells are critical to the creation and maintenance of pain facilitation through the release of proinflammatory cytokines. Adenoviral vectors encoding human IL-10 (AD-h-IL10) blocked and reversed pain facilitation [45]. Another cytokine gene, IL-2, can be delivered both by adenoviral vectors or plasmids, both of which lead to obvious antinociceptive effects [46, 47]. This effect could be blocked by naloxone, illustrating the relationship between IL-2 and opioid receptors [47]. Previous studies have demonstrated that IL-2 can suppress afferent sensory transmission and act as Ca^2+^ channel blockers, but the mechanism of IL-2-induced antinociception is still unclear [46]. The effects of AV vector-mediated gene transfer in pain models are summarized in Table 3.

## 5. Lentivirus (LV)

Lentiviral vectors have the advantages of long-term transgene expression, low immunogenicity, and the ability to accommodate larger transgenes [48]. LVs belong to a subclass of retroviruses that integrate into the host cell genome. Due to their natural integration ability, LVs have a lot of potential in central nervous system applications. LVs have been extensively utilized for ex vivo gene transfer because of their strong tropism for neural stem and progenitor cells [29]. Single microinjection of lentiviral vector-mediated intraspinal gene transfer allowed for the diffusion of vectors along the rostrocaudal axis, though expression was still restricted to the gray matter of the ipsilateral dorsal spinal cord. In addition, transgene expression in glial cells did not modify glial activity or alter animals' locomotor behavior [49].

Lentiviral vectors are efficient tools to induce sustained expression of trophic factors in specific areas of the central nervous system (CNS). Glial cell line-derived neurotrophic factor (GDNF), which regulates neuronal survival and gene expression, is expressed in both the central and peripheral nervous systems. Reduction in GDNF and its receptor levels in the nociceptive afferent system may contribute to the development and maintenance of neuropathic pain states [50]. Intraspinal administration of lentiviral vectors expressing GDNF lead to a large and sustained expression of transgenes in both neurons and glial cells. Gene delivery of GDNF via lentiviruses produced a partial but significant reversal of thermal and mechanical hyperalgesia [51].

Nuclear factor *κ*B (NF-*κ*B) is a pleiotropic factor involved in transcriptional control of some inflammation and pain-related molecules. In a model of chronic constriction injury (CCI), lentiviral vector-mediated overproduction of I*κ*B*α*, which is a natural endogenous inhibitor of NF-*κ*B, inhibited NF-*κ*B activity in glial cells of the dorsal spinal cord and produced prolonged antihyperalgesic and antiallodynic effects. I*κ*B*α* overproduction also reduced expression of interleukin-6 (IL-6) and inducible nitric oxide synthase (iNOS), suggesting that I*κ*B*α* may relieve pain via the prevention of CCI-associated expression of IL-6 and iNOS [52].

## 6. Human Foamy Virus (HFV)

Human foamy virus, the first identified human retrovirus, is nonpathogenic and has several unique features related to gene transfer, making it a promising vector system for gene therapy [53]. The potential advantages of FV vectors include a broad host range, the largest packaging capacity of any retrovirus and the ability to persist in quiescent cells [54, 55]. Because of these features, foamy vectors have the unique potential to safely and efficiently deliver several genes into a number of different cell types in vivo [54] and are especially useful for transducing hematopoietic cells. They have been demonstrated to mediate efficient and stable gene transfer into hematopoietic stem cells (HSCs) in mouse and canine animal models, which indicates that they have a unique integration profile and suggests they may be safer than gammaretroviruses or lentiviral vectors [55].

Liu et al. found that gene transfer using a human foamy virus (HFV) vector can reduce below-injury level mechanical allodynia and thermal hyperalgesia after spinal cord injury (SCI). In that study, the glutamic acid decarboxylase (GAD) gene was transferred into dorsal root ganglion (DRG) cells using a novel HFV vector that expresses GAD (vector rdvGAD67) for 7 days after T13 spinal cord hemisection to achieve release of gamma-aminobutyric acid (GABA). Subcutaneous inoculation of a replication-defective HFV vector attenuated pain evoked by SCI and also enhanced the production of GAD and tonic GABA release from transduced DRG neurons [56].

## 7. Possible Mechanisms of Viral Vector-Mediated Gene Therapy for Chronic Pain

**Figure 1:**
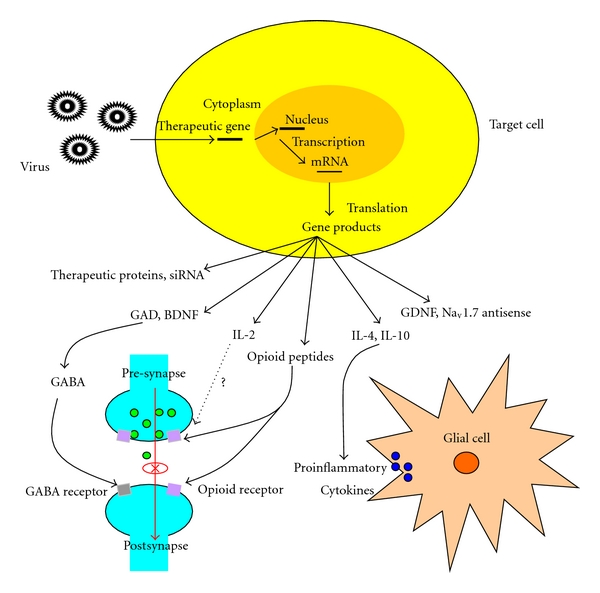
Possible mechanisms of viral vector actions in gene therapy of chronic pain. Recombinant viral vectors encoding therapeutic genes infect target cells and express antinociceptive substances after subcutaneous inoculation or intrathecal administration. Gene products such as GAD and BDNF can lead to the release of GABA, which is an inhibitory neurotransmitter. IL-4 and IL-10 can suppress the expression of proinflammatory cytokines produced by activated glial cells and antagonize the signaling pathway activated by these cytokines. Endogenous opioid peptides have analgesic effects through opioid receptors. Na_v_1.7 antisense can prevent an increase in Na_v_1.7 expression and decrease inflammatory hyperalgesia.

Studies have showed that viral vector-mediated therapeutic genes play significant analgesic effects in several different pain models. Understanding the mechanism of viral vector function in the gene therapy of chronic pain will help us to improve treatment efficacy and search for new therapeutic strategies. The mechanisms of viral vector-based gene therapy involve several pathways (Figure 1). Some gene products, such as GABA, can block nociceptive neurotransmission at the first synapse between the primary peripheral nociceptor and the second order neuron in the spinal cord. Anti-inflammatory cytokines expressed by viral vector-mediated therapeutic gene can reduce central neuroimmune activation. Antisense or RNA sequences enact their analgesic effect by reducing the expression of gene products essential to the development of chronic pain [2]. 

Activation of nociceptive primary neurons leads to central release of excitatory neurotransmitters or neuromediators, such as glutamate, substance P, and ATP, which are all related to pathological pain [43]. Inhibitory interneurons and descending modulatory control systems are dysfunctional after nerve injury, leading to disinhibition or facilitation of spinal cord dorsal horn neurons and to further central sensitization [57]. Spinal cord glial cells are activated via neuronal chemokines, neurotransmitters, and substances released by damaged neurons [58]. Activated glial cells further enhance neuronal excitability by releasing cytokines, especially proinflammatory cytokines, thus increasing the concentration of glutamate and the downregulation of GABA receptor function [57, 58].

Proinflammatory cytokines (TNF-*α*, IL-1*β*, IL-6, etc.) enhance pain expression by increasing the excitability of dorsal horn pain transmission neurons and upregulating AMPA and NMDA receptors [45]. Anti-inflammatory cytokines (IL-4, IL-10) are known to suppress the production of proinflammatory cytokines that are released by activated spinal cord glial cells. It has been shown that viral vectors encoding anti-inflammatory cytokines can block and reverse pain facilitation in various pain models. Hao et al. demonstrated that HSV-mediated expression of IL-4 reduced c-Fos expression in the dorsal horn in the SNL model of neuropathic pain, decreased levels of IL-1*β* and PGE2 in the dorsal horn and reduced the phosphorylation of spinal p-p38 [23].

Endomorphin-2, *β*-endorphin, and proenkephalin, which are all endogenous opioid peptides, are often encoded in viral vectors because of their analgesic effect. Endogenous opioid peptides evoke the analgesic effect by stimulating opioid receptors. These receptors can be found in the central and peripheral terminals of primary nociceptors, second-order neurons in dorsal horn of spinal cord and other tissues such as brain [59]. Transduction of other genes, such as GAD and BDNF, via viral vector-mediated gene transfer can increase the release of GABA to relieve pain [46, 51]. Naik et al. speculated that GABA-mediated depolarization influences the excitability of sensory neurons both in cell bodies and nerve terminals by inactivating other voltage-sensitive channels, such as Ca^2+^ and Na^+^. It is also possible that the GABA-activated Cl^−^ current directly inhibits ATP-evoked excitatory currents in DRG neurons [60].

## 8. Prospects

Viral vector-mediated gene transfer has been applied in some clinical trials to evaluate its safety and tolerance. In 2002, a phase I study of replication-competent adenovirus-mediated double suicide gene therapy of prostate cancer demonstrated that this treatment could be safely applied to humans [61]. Other clinical trials of viral vectors for gene therapy have also been shown to be safe and well tolerated, but the effect of this treatment was not very significant [62, 63]. For chronic pain, the first human trial of gene therapy began enrolling subjects in December 2008. This was a phase 1 study of a nonreplicating herpes simplex virus- (HSV-) based vector that expressed preproenkephalin in patients with intractable pain from cancer [64]. These clinical trials promote the development of gene therapy for chronic pain.

 Viral vectors for gene therapy are currently mainly in preclinical trials. Although gene therapy is a promising new treatment and has some advantages over other therapies, there are still several problems left to be solved. Transfection efficiency, specific targeting, and the safety of viral vectors need to be improved. Potential mutations, antiviral response and total inefficacy also limit the application of gene therapy. Achieving a restricted, local production of therapeutic transgenes may decrease some adverse reactions. Previous studies focused on the application of single therapeutic gene, while codelivering antagonizing genes may decrease the side effects and improve efficacy. For instance, Xu et al. demonstrated that recombinant AAV-mediated transgene expression of *μ*-opioid receptor which targets specifically DRG neurons could avoide undesired systemic effects of opioids and also enhance the antinociceptive effects of morphine [33]. The development of biomedical technologies will lead to the construction of safer and more efficient viral vectors. It is believed that successful application of viral vectors for gene therapy could lead to a breakthrough in the treatment of chronic pain and other diseases.

## Figures and Tables

**Table 1:** Analgesic effect of HSV vectors encoding therapeutic genes in pain treatment.

Pain models	Gene product	Inoculation	References
Acute pain	Preproenkephalin	To the skin of dorsal hindpaw	Wilson et al. [16]
Inflammatory pain	Preproenkephalin A	Infected on scarified hind footpads	Braz et al. [17]
Neuropathic pain	Proenkephalin A	Unilateral peripheral inoculation	Meunier et al. [21]
Cutaneous hyperalgesia	Preproenkephalin	Subcutaneous inoculation	Yeomans et al. [9]
Bladder hyperactivity and pain	Preproenkephalin	Injected into the bladder wall	Yokoyama et al. [20]
Inflammatory pain	Endomorphin-2	Subcutaneous inoculation	Hao et al. [18]
Neuropathic pain	Endomorphin-2	Subcutaneous inoculation	Wolfe et al. [19]
Neuropathic pain	IL-4	Subcutaneous inoculation	Hao et al. [23]
Neuropathic pain	sTNFRs	Subcutaneous inoculation	Peng et al. [24]
Neuropathic pain	GAD67	Subcutaneous inoculation	Hao et al. [25]
Neuropathic pain	GAD67	Subcutaneous inoculation	Liu et al. [26]
Inflammatory pain	Na_v_1.7 antisense	Subcutaneous inoculation	Yeomans et al. [27]
Cancer pain	Proenkephalin	Subcutaneous inoculation	Goss et al. [14]

**Table 2:** Analgesic effect of AAV vectors encoding therapeutic genes in chronic pain treatment.

Pain models	Gene product	Inoculation	References
Neuropathic pain Neuropathic pain Neuropathic pain Inflammation pain Neuropathic pain	IL-10 shGCH1 Prepro-*β*-endorphin *μ*-opioid receptor BDNF	Intrathecal administration Injected into sciatic nerve Intrathecal administration Injected into DRG Injected into the dorsal horn	Milligan et al. [31] Kim et al. [32] Storek et al. [34] Xu et al. [33] Eaton et al. [35]

**Table 3:** Analgesic effect of AV vectors encoding therapeutic genes in chronic pain treatment.

Pain models	Gene product	Inoculation	References
Inflammatory pain Pathological pain Inflammatory pain Neuropathic pain	GAD65 IL-10 *β*-endorphin Interleukin-2	Injected into the left trigeminal ganglion Intrathecal administration Intrathecal administration Intrathecal administration	Vit et al. [43] Milligan et al. [31] Finegold et al. [44] Yao et al. [46]
